# Child emotional and behavioral difficulties and parent stress during COVID-19 lockdown in Sri Lankan families

**DOI:** 10.1371/journal.pone.0271757

**Published:** 2022-08-03

**Authors:** Ashan Athapathu, Deluckshi Navaratnam, Minul Doluweera, Guwani Liyanage

**Affiliations:** 1 Faculty of Medicine, University of Moratuwa, Moratuwa, Sri Lanka; 2 Lady Ridgeway Hospital for Children, Colombo, Sri Lanka; 3 Faculty of Medicine, University of Colombo, Colombo, Sri Lanka; 4 Department of Paediatrics, Faculty of Medical Sciences, University of Sri Jayewardenepura, Nugegoda, Sri Lanka; Institute of Physiology and Basic Medicine, RUSSIAN FEDERATION

## Abstract

**Introduction:**

Understanding parents’ and children’s mental health issues would help design population-specific intervention programs. The present study explored parents’ perceived stress and child emotions and behavior during the COVID-19 lockdown among Sri Lankan families.

**Methods:**

A cross-sectional survey was conducted among Sri Lankan parents of children aged 11 to 17 years. Validated instruments (Perceived Stress Scale-PSS and Strengths and Difficulties Questionnaire-SDQ) evaluated parental stress, child emotions, and hyperactivity/inattention. Multiple linear regression assessed the predictors of mental health issues, including the interaction between age and gender.

**Results:**

Three hundred fifty-five parents responded to the survey (mothers:76%). One-third of parents experienced difficulties with their children during the pandemic. Emotions and hyperactivity-inattention problems measured via the SDQ scale were high among 38% of children, while the perceived stress was high in 79.2% of parents. Overall, child emotions and hyperactivity-inattention increased with decreasing age, increasing parent stress, having middle-income compared to high-income, and having a family member/close relative tested positive for COVID-19. Hyperactivity-inattention (29.3%) was more than the emotional problems (22%) among children. The emotional problems were reported more with increasing parent stress, while child hyperactivity-inattention alone was reported more with decreasing age, middle-income compared to high-income families, and increasing parent stress. Also, the interaction effect of age and gender indicated that higher age was related to greater parent-reported hyperactivity-inattention problems in males.

**Conclusions:**

The findings highlight how the COVID-19 crisis and social isolation have contributed to increased parental stress and child emotional and hyperactivity-inattention problems. In addition to cautioning the healthcare workers, socio-culturally appropriate preventive and supportive mental health programs may help deal with further waves of COVID-19 or any other adverse circumstances.

## Introduction

During the COVID-19 pandemic, free movement and lifestyles were disrupted because of home isolation, school closures, and loss of jobs. The impact was more for heavily tourism-reliant economies (e.g., Sri Lanka) [1]. Families had to cope with unprecedented and unusually new situations. Parents were managing the care and education of children while juggling with changes in their work styles. People’s free movement was restricted; thus, social interactions beyond the nuclear family unit became primarily virtual. In addition, a rise in violence and conflict at home fronts was frequent [2]. Children were the worst affected, impacting their school education and extracurricular activities [1]. Most importantly, children were deprived of their socialization and play spaces.

Mental health well-being had hardly been explored during social isolation or home confinement before the pandemic. However, since the beginning of the COVID-19 pandemic, several reports have been published on the psychological well-being of parents and children during social isolation and lockdown [3–5]. In Italy, parents of children aged 3–13 years showed very high rates of psychological distress during the lockdown [4]. Soriano-Ferrer et al., who investigated children with dyslexia using a pre-post design, reported a negative impact on psychological wellbeing during quarantine compared to pre-quarantine conditions among children with dyslexia [6]. Marchetti et al. described an association of parents’ psychological distress with hyperactivity/inattention in children during the pandemic [5]. Further, they showed that parent verbal hostility and child emotional problems positively mediate this relationship. Another important phenomenon during the COVID-19 outbreak is the so-called parental-related exhaustion, defined as feelings of being overextended and depleted of one’s emotional and physical resources. Exhaustion is a key aspect of parental burnout, which results from prolonged exposure to parental stress, which influences the psychological wellbeing of children [7, 8].

Much research has explored potential risk factors associated with mental health wellbeing among children, adolescents, and parents during the pandemic. However, there are no data on the role of individual and COVID- 19 contextual variables on parental stress and children’s psychological wellbeing in the Sri Lankan context. The pandemic could negatively impact children and adolescents living in poor socioeconomic conditions [9]. These children had less opportunity to study from home and access mental health services, increasing preexisting social and learning gaps. Also, preexisting mental health conditions or disabilities and having relatives or friends tested positive for COVID-19 have increased psychological distress among children and adolescents [9]. In a study on parental-related exhaustion during the COVID-19 pandemic, Marchetti et al. found that parenting-related exhaustion was predicted by psychological distress, lower parental resilience, motherhood, fewer perceived social connections, and being single, as well as having a child with special needs, having a large number of children, and having younger children [5].

The pandemic’s impact on children’s mental health and parental stress in Sri Lanka is only anecdotal; no studies have explored psychological wellbeing during this period so far. Moreover, during the pandemic and even in pre-pandemic times, children’s psychological well-being on parent stress and vice versa had hardly been explored in the Sri Lankan context [10, 11].

Despite Sri Lanka being a low-middle income country, it is believed that the existing robust healthcare system could respond to many adverse circumstances related to mental and physical health [12]. Also, Sri Lankan culture, family relationships, and community support may buffer these effects. However, the impact of prolonged lockdowns and school closures during the COVID-19 pandemic was unique and unprecedented. It was invariably a big challenge for any healthcare system. Therefore, understanding the impact of the pandemic on mental health is important for policymakers and clinicians to support families during any crisis. Also, the findings would fill the gap in knowledge related to this topic from the perspective of a low-middle-income country. Hence, this study was carried out to explore the burden of children’s mental health problems and parent stress in the worst affected district in Sri Lanka during the COVID-19 pandemic. Moreover, we aimed to investigate individual (e.g., educational attainment) and contextual factors (e.g., having a relative or family member with COVID-19 infection) associated with psychological wellbeing.

## Materials and methods

### Participants

This survey was commenced in October 2020, approximately seven months into the Covid-19 pandemic, amidst a prolonged lockdown and closure of schools. Online data collection was continued for two months. The participants were parents/caregivers of 11 to 17-year-old children from the Colombo District. Only children without physician-diagnosed significant physical disorders (viz. chronic kidney disease, chronic liver disease), neurodevelopmental disorders (viz. attention-deficit/hyperactivity, autistic spectrum disorder, learning disabilities, intellectual disability, cerebral palsy, and impairments in vision/hearing) and children/parents without psychiatric illnesses were included. Only one child from the given age group was recruited from one family. In sample calculation, the expected population proportion with emotional and behavioral difficulties was taken as 0.43 [13] with a confidence interval of 95% (z = 1.96) and a level of precision of 0.05. The number of non-respondents was predicted to be 10%, added to the desired sample size. The final calculated sample size was 323. Ethical approval for this study was granted by the Ethics Committee of Sri Lanka College of Pediatricians (SLCP/ERC/2021/12). The study followed the guidelines of the Declaration of Helsinki. Informed written consent was taken from the parent/guardian before data collection.

### Study instruments

The main survey instrument consisted of three sections: 1. Basic characteristics of the participants relevant to the study 2. Strengths and Difficulties Questionnaire (SDQ) [14] and 3. Perceived Stress Scale [15] (PSS). The first section of the questionnaire was piloted among parents to test the clarity and verify its overall comprehensibility. In addition to socio-demography-related questions, the first section included factors that might influence children’s behaviors and parent stress, such as working pattern, employment, single parent/two-parent family, extended family support, and having a family member or relative tested positive for COVID-19 infection. The SDQ evaluates children’s emotional problems, conduct problems, hyperactivity-inattention, peer problems, and prosocial behavior. The emotional problems scale (five questions) and the hyperactivity-inattention scale (five questions) were used for the current study. Conduct problems and peer problems scales have questions related to school and peers. Therefore, those scales were not used for the current study due to the school closure with the lockdown. The PSS, a previously validated tool, includes ten questions asking how life has been experienced as unpredictable, uncontrollable, and overloaded during the previous month.

### Definitions

Education was classified as follows; less than secondary (Grade 11 and below), secondary (up to grade 13), post-secondary-non-tertiary, and tertiary (had done programs with specific professional qualifications). Household income was categorized into low: <45,000 Sri Lankan rupees (LKR), middle:45,000–120,000 LKR and high: >120,000 LKR [16].

### Procedure

An online survey link was disseminated to the participants through selected schools in the Colombo district using an online platform. Additional participants were enrolled using the snowball sampling method to achieve the required sample size by asking respondents to share the survey link among their contacts. We restricted the survey to the Colombo district as it had the heaviest toll of COVID-19 infection and a lengthy lockdown. The recruitment phase included screening, obtaining informed consent, and the survey questionnaire. The screening questionnaire obtained the child’s age, illnesses/disabilities of parent or child, and the district they were living in. Subsequently, eligibility was assessed based on inclusion and exclusion criteria. After going through the information sheet with the study aims and procedure, the respondents provided consent. Those who consented proceeded to complete the questionnaire. They could withdraw at any time, and the data was anonymous.

### Data analysis

The emotional and hyperactivity-inattention scale of SDQ was measured on a three-point Likert scale (not true = 0, somewhat true = 1, and certainly true = 2). The total score for each scale was ten. The total score for SDQ was categorized into original 3-bands (Emotional problems score, normal: 0 to 3, borderline: and abnormal:5 to10, and hyperactivity-inattention score, normal:0 to 2, borderline:3 and abnormal:4–10). These bandings were defined based on a population-based UK survey since local cut-off points were unavailable [17].

The Cronbach alpha coefficient for the ten items of SDQ (emotional and hyperactivity-inattention) from the present study was 0.75. The coefficients for the emotional problem and hyperactivity-inattention scores were 0.72 and 0.62. In a validation study, Cronbach alpha coefficients for the emotional problem scores and hyperactivity-inattention scores were 0.67 and 0.77, respectively [17]. A five-point Likert scale was used to score the PS scale (never = 0, almost never = 1, sometimes = 2, often = 3, and very often = 4). The individual score was calculated out of 40 marks. PS score was categorized into three levels (low stress = 0–13, moderate stress = 14–26 and high stress = 27–40). Cronbach alpha from the present study was 0.70 for ten items of PSS. Cohen et al. reported a Cronbach alpha of 0.78 [18]. Spearman correlation tested the association between SDQ score and PSS score. All potential covariates associated with SDQ (log-transformed) were analyzed using univariate regression to examine which children are at higher risk of being particularly impacted. All variables with a p-value <0.05 and variables with a-priori knowledge were considered in multivariate regression analysis (gender of the child) [19, 20]. Subsequently, the effect of interaction between age and gender was also explored. Similarly, the predictors of emotional problem score and hyperactivity-inattention score were analyzed with linear regression separately. All independent variables, including the interaction variable, were checked for multicollinearity. Residual and scatter plots were used for assumptions of normality and homoscedasticity.

## Results

A total of 365 eligible respondents participated in the survey. Twenty-one were excluded due to incomplete questionnaires. The response rate was 94%. Mother was the respondent in the majority (76%). A smaller fraction (5.1) were single parents. Most were nuclear families (59%). Almost half had a high income. Sixty-six percent (n = 124) of the respondents were employed in an income-generating activity (Table 1). Of them, sixty-two respondents experienced a wage reduction (n = 58) or loss of employment (n = 4) during the pandemic. Loss of employment/wage reduction was not significantly different between income groups (*X*^2^ = 6.2, df = 2, p = 0.05). Sixty-three respondents believed they had more savings during the pandemic than before; the high-income group had more savings than the other two income groups (*X*^2^ = 23.8, df = 2, p = <0.001).

**Table 1:** Basic characteristics of the study population.

Characteristic	n (%)
*Age of child, mean (SD)	13.57 (1.98)
Gender of the child, male	211 (59.4)
Respondents’ education	
Less than secondary	25 (07)
Secondary	56 (15.8)
Post-secondary non-tertiary	94 (26.5)
Tertiary	180 (50.7)
Employment (Income generating)	248 (70)
Lost job/wage cut	61 (17.2)
More savings than before	63 (17.7)
Household income (LKR)	
Low	97 (27.3)
Middle	81 (22.8)
High	177 (49.9)
Family/relative tested positive for COVID-19	69 (19.5)
*SDQ total score, median (IQR)	6 (3, 9)
SDQ-Emotional problem score	
Normal	277 (78)
Borderline	29 (8.2)
Abnormal	49 (13.8)
SDQ-Hyperactivity/inattention score	
Normal	251 (70.7)
Borderline	45 (12.7)
Abnormal	59 (16.6)
*Perceived stress score, mean (SD)	17.6 (5.9)

*Expressed as mean (SD) or median (IQR)

Abbreviations: IQR-Interquartile range, LKR-Sri Lankan rupees, SD-Standard deviation

**Fig 1:**
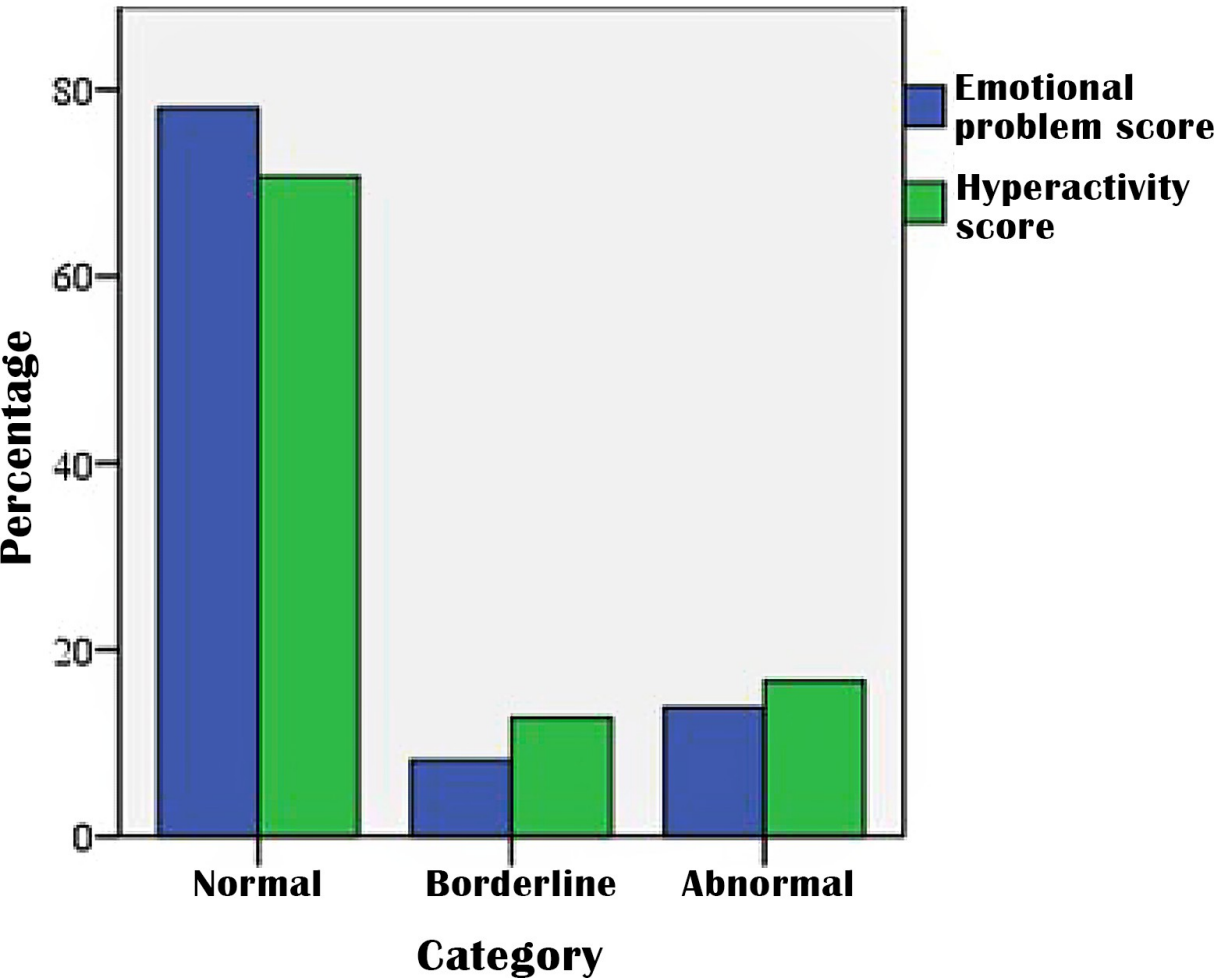
Emotional problem score and hyperactivity-inattention score.

Thirty-seven percent of parents experienced more difficulties with their children during the pandemic than before. The mean (SD) of the parent-reported SDQ score was 6.35(3.8). The total score was borderline/high among 38% of children, while emotional and hyperactivity-inattention scores were borderline/abnormal in 22% and 29.3%, respectively (Fig 1). Spearman correlation revealed a moderate but significant association between SDQ and PS scores (r = 0.39, p = <0.001).

**Fig 2:**
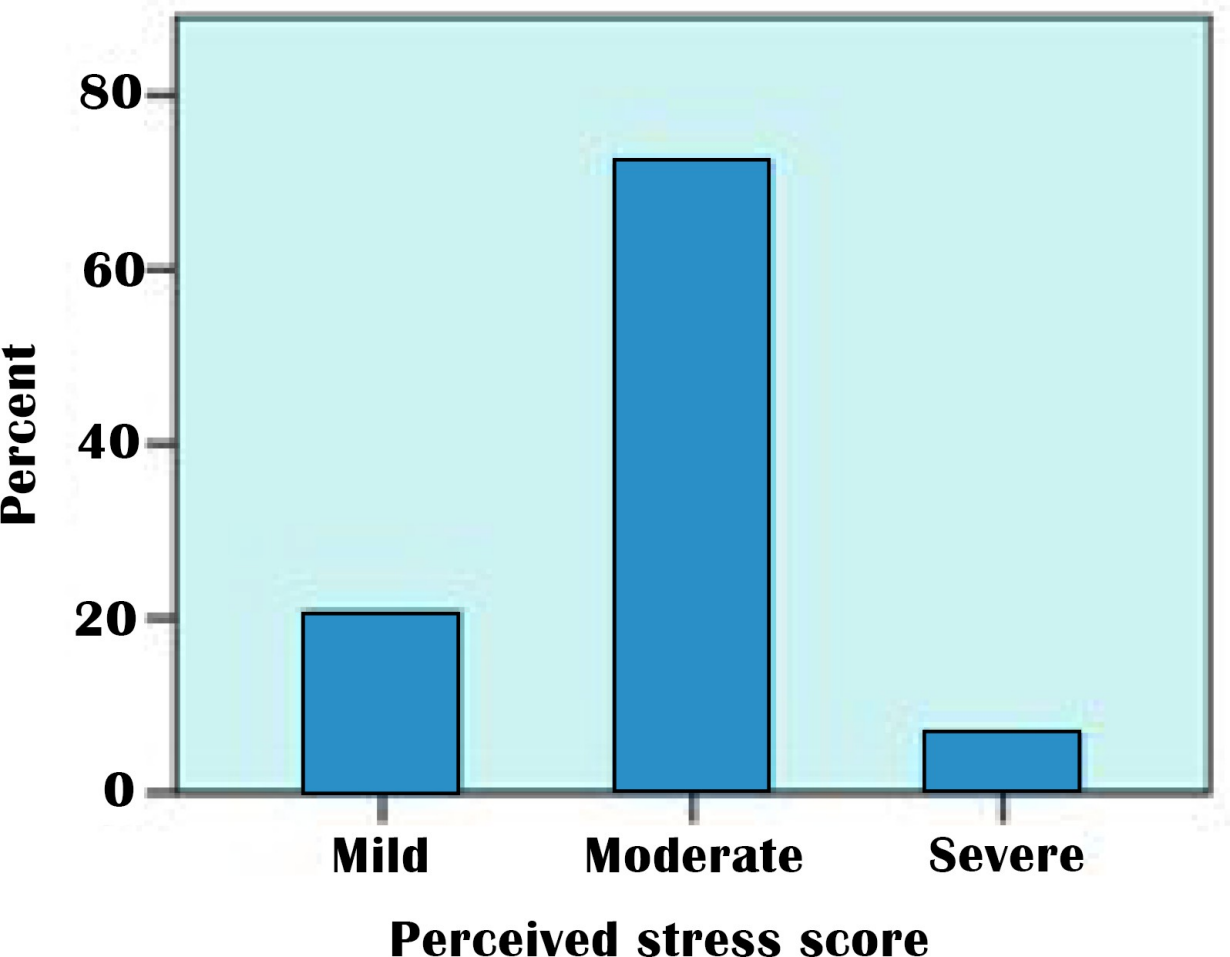
Perceived stress score of the sample population.

The mean perceived stress score of the respondent was 17.6(5.9). When categorized, the PS score was high, moderate, and low at 6.8%, 72.4%, and 20.8%, respectively (Fig 2). The bivariate analysis showed that having a family/close relative positive for COVID-19 infection (OR:2.36, 95% CI:0.820,3.893, p = 0.003) and being a female (OR: -1.76, 95%CI: -3.004, -0.509, p = 0.006) were risk factors for increased parent stress. Having extended family (p = 0.09) or income level (p = 0.33) did not significantly impact parent stress.

A simple linear regression analysis was performed to assess the factors associated with the SDQ score (Table 2). The child’s age, having a family member or close relative tested positive for COVID-19, household income, and perceived stress score were significantly associated with the SDQ score.

**Table 2:** Unadjusted linear regression analysis for predictors of the log transformed SDQ.

Variable	β	95% CI		p
Age of child	-0.69	-0.111	-0.027	0.001
Gender of the child (male)	-0.07	-0.241	0.102	0.42
Gender of parent (male)	-0.17	-0.364	0.030	0.10
Single parent (yes)	-0.15	-0.529	0.239	0.46
Respondents’ education				
Degree level or higher	Ref			
Less than collegiate	0.25	-0.084	0.574	0.14
Collegiate	0.15	-0.082	0.380	0.21
Vocational	0.13	-0.323	0.058	0.17
Employment (Homemaker)	0.00	-0.176	0.181	0.98
Household income (LKR)				
High	Ref			
Middle	0.23	0.031	0.430	0.02
Low	-0.07	-0.255	0.123	0.49
*Tested positive	0.38	0.171	0.587	<0.001
PS score	0.05	0.035	0.062	<0.001

*Family/relative tested positive for COVID-19 infection. Abbreviation: CI—Confidence interval, LKR-Sri Lankan rupees, PS-Perceived stress

In multivariate regression analysis, an increase in the child’s age by one year decreased the log transformed SDQ by 0.11 points (Table 3). Compared to high-income, middle-income families reported higher SDQ scores among children. On the other hand, having family/relatives tested positive for COVID-19 and perceived stress were positively associated with log_10_ SDQ. One point in perceived stress score increased the log_10_ SDQ by 0.06 points. The highest contribution to the model was from having family/relatives tested positive for COVID-19. Subsequently, age and gender interaction variables were explored. It showed a significant improvement in the model for the hyperactivity-inattention score. Higher age was related to greater parent-reported hyperactivity problems in males.

**Table 3:** Multiple linear regression analysis of predictors of the log_10_ SDQ total, log_10_ emotional problem score, and log_10_ hyperactivity-inattention score.

Variable	Log_10_ SDQ score (Total)				Log_10_ emotional problem score				Log_10_ hyperactivity-inattention score			
	Β	95% CI		*p*	β	95% CI		*p*	β	95% CI		*p*
Age of child	-0.11	-0.192	-0.033	**0.006**	-0.04	-0.070	0.010	0.57	-0.05	-0.077	-0.029	**<0.001**
Gender (male)	-0.01	-0.082	0.077	0.95	-0.01	-0.039	0.032	0.84	0.01	-0.019	0.029	0.69
Age*male	0.06	-0.017	0.141	0.13	-0.04	-0.142	0.001	0.05	0.34	0.010	0.058	**0.005**
Income												
Middle	0.22	0.018	0.417	**0.03**	0.44	-0.041	0.128	0.31	0.06	0.002	0.122	**0.04**
Low	0.02	-0.175	0.201	0.88	-0.55	-0.138	0.029	0.20	0.03	-0.025	0.089	0.27
^a^Tested positive	0.30	0.094	0.503	**0.004**	0.66	-0.017	0.149	0.12	0.06	-0.004	0.118	0.07
PS score	0.06	0.039	0.070	**<0.001**	0.01	0.004	0.019	**<0.002**	0.12	0.008	0.017	**<0.001**

Model for total SDQ score: (F 6,341 = 14.3; p = 0.001; R^2^ = 21%)

Model for emotional problem score: (F, 337 = 5.4; p<0.001; R^2^ = 13%)

Model for hyperactivity-inattention score: (F 6,340 = 11.4; p<0.001; R^2^ = 18%).

^a^Family member/close relative tested positive for COVID-19 infection

Abbreviations: CI—Confidence interval, PS-Perceived stress

## Discussion

To our knowledge, this is the first study exploring psychological well-being in children and adolescents in Sri Lanka during the COVID-19 pandemic. We have also investigated the relationship between paternal stress and a child’s emotional problems, hyperactivity-inattention, and potential individual and contextual factors associated with mental health issues in this population. We believe these findings could be utilized to improve clinical practice, preventive programs, and health policies to provide suitable support in the present crisis and comparable future situations.

The proportion of emotional problems and hyperactivity-inattention among children and adolescents in our study was similar to many other studies reported worldwide during the pandemic [9, 21]. Hyperactivity-inattention predominated over emotional problems. Home confinement with a lack of free space and absence of organized educational activities during prolonged lockdowns may have impacted their behavioral functioning (viz. hyperactivity-inattention) than emotional wellbeing.

More than one-third of the participants reported that they experienced more difficulties with their children during the pandemic compared to the pre-pandemic period. Also, the results showed that parental stress was increased during the lockdown period, consistent with reports from other parts of the world [9, 13]. Parents had to face many challenges during the unprecedented and completely new situation. Parents were left alone in their children’s education and learning while managing house chores and job activities.

We noted that the child’s age was a significant predictor of the total SDQ score; younger children were more affected than the older children. Age was not an independent predictor of emotional problems, yet hyperactivity-inattention was reported more among younger children than older children. Although Christener et al. reported similar results, older children (7–10 years) had more emotional symptoms and fewer conduct problems/hyperactivity than younger children (3–6 years) [22]. As previously shown, younger children expect more parents’ engagement than older children [5, 9]. However, parent engagement was probably less during the pandemic. They were likely to be more worried about their family’s finances, and physical health, than engaging and interacting with their children. Also, older children are generally more independent than younger children; they have better opportunities to contact their friends or relatives using electronic devices, which keeps them occupied during the lockdown [23].

In the present study, gender and age interaction revealed that increasing age was a risk factor for male hyperactivity-inattention problems. However, gender alone did not impact the SDQ scores except for the interaction effect. Previous research has reported a male preponderance in mental health issues [5]. Also, males are more likely to externalize emotions, which leads to aggressive, impulsive, coercive, and noncompliant behavior than females [24].

Surprisingly, reported mental health problems in children in the low-income group were not significantly different from middle- and high-income families. However, the middle-income group reported higher hyperactivity/inattention among children than the high-income group. Many middle-income families desire to keep up with societal trends [25]. In turn, the children of those families may suffer the greatest disruptions to mental health when parents may not be able to support their desired activities financially due to hardships during the pandemic. On the other hand, securing spendable funds for essentials for low-income families remains their most important concern for the parents. The children of those families are more resilient to acute changes, possibly through repeated exposure to hardships. Therefore, living in disadvantaged families per se is a protective factor against adversities [26]. However, further research with composite indices of socioeconomic status may help clarify these connections.

Overall, having a family member or a close relative with COVID-19 infection appeared to significantly predict children’s hyperactivity/inattention and total SDQ scores. Further, it also increased the perceived stress score reported by parents significantly. The stigma and uncertainties related to and worrying about getting infected may have contributed to the increased psychological issues among parents and children [27]. Reported stress was higher among mothers than fathers. Historically, mothers have been the primary caregivers, and fathers’ role has been mainly getting resources and services to the family. However, the trend is changing; more and more fathers are involved in active childcare [28]. In addition, it has been shown that Asian families have multiple important caregivers (e.g., extended family) functioning in the child’s everyday environment. However, in our study, the extended family had no significant impact on reducing parent stress. The extended family could help with household chores and childcare and lessen the burden on the parents [29]. However, extended families living together in crowded households may cause family conflicts and increased parental distress [30]. Therefore, the relationship between extended family and parental stress could be complex during a pandemic. The actual effects of the extended family may differ based on various household, socioeconomic and cultural factors. Therefore, it may need further evaluation in the Sri Lankan context. These factors put parents at a higher risk of experiencing distress, possibly weakening their ability to be supportive caregivers.

In the present study, parents’ perceived stress was a significant independent risk factor for a child’s overall SDQ score and emotional and behavioral domains. Parents’ perceived stress was the only significant independent risk factor for emotional problem scores. Therefore, it is critical to foster a sense of calm and security among caregivers and provide guidance to balance immense challenges and pressures during the pandemic. However, despite previous studies reporting parents’ perceived stress as a risk factor for a child’s emotional and behavioral problems [13, 19, 23], the reverse had been proposed by a few other studies [31]; that is, a child’s behavior triggering parent stress. Therefore, a bidirectional relationship between parents’ perceived stress and a child’s emotional and behavioral issues is likely. If so, the interventions should be targeted at both variables.

The following strengths and limitations should be considered when interpreting the study findings. As for strengths, there are hardly any published reports on this topic in Sri Lanka, particularly during a crisis. We used validated measures through an online survey and enrolled a wide sample from the most affected district in the country. Thus, it provides helpful information for clinicians/social workers and policymakers to understand the gravity of the problems related to child mental health and parent stress during catastrophes. All health care professionals and institutions should increase awareness of this pandemic’s adverse effects on children and adolescents. Health authorities should allocate adequate resources, establish mental health promotion and intervention programs to support vulnerable children and adolescents, and prepare for possible further waves of COVID-19 or similar crises. This study suggests how clinicians should implement interventions to support families to improve the mental well-being of children based on age, sex, and socioeconomic status. Advising parents on how parents stress could negatively affect a child’s mental well-being and empowering them to manage stressful situations are essential strategies. Also, additional support could be provided for families with COVID-19 infection via field primary healthcare workers. Discussing fears and the negative emotions related to the pandemic would be valuable for families’ well-being. Parents can be advised to educate the children about the pandemic so that children will be more self-confident and ready for positive adjustment. Also, guidelines should be formulated to support children and parents in coping with the pandemic while developing resilience. This study also encourages the researchers for follow-up studies to evaluate the long-term impact of the pandemic on mental health.

As limitations, firstly, although we identified risk factors of psychological distress in children, we cannot attribute direct causality due to the cross-sectional nature of this study. Second, a selection bias in sampling is possible as only the subjects with internet access could participate. Third, the sample was not very well-balanced in the gender of the parent; in the vast majority, the respondent was the mother. Therefore, caution should be exercised when interpreting the effects of parent gender. Fourth, we relied on reported data, and the existence of a social desirability bias could not be avoided. Finally, since parent stress and child mental health issues were assessed with a parent-report questionnaire, common method variance was a concern. For example, stressed-out parents may overestimate the child’s emotional and behavioral status and consequently over-report.

## Conclusions

The findings of our study help understand the child’s emotional and behavioral issues and parents’ stress in a resource-poor setting during the COVID-19 lockdown. The clinicians and social workers should be cautioned about mental health issues and the associated individual, family, and contextual factors. In addition, socio-culturally appropriate preventive and supportive programs should be designed to deal with further waves of COVID-19 or any other future adverse circumstances.

## Supporting information

S1 File (PDF)
