# Supplementary material for: Child emotional and behavioral difficulties and parent stress during COVID-19 lockdown in Sri Lankan families
Source: PLoS One. 2022 Aug 3;17(8):e0271757. doi: 10.1371/journal.pone.0271757 (PMC9348699; doi:10.1371/journal.pone.0271757)
Supplement: S1 File — (PDF) [file pone.0271757.s001.pdf]

5th March 2021 Ref. No. SLCP/ERC/2021/12

This communication is from the ***Ethics Review Committee*** of the Sri Lanka College of Paediatricians

Chairman:
Dr. G.N.Lucas

Members:
Professor D.G.Harendra De Silva
Dr. B.J.C.Perera
Dr. Rasika Gunapala
Dr. Piyusha Atapattu
Professor Shaman Rajindrajith
Professor Dulanie Gunasekera
Dr. Dharmasri Jayawardhene
Dr. Ruwanthi Perera
Mr. Dharmathilake
Ms. Priyadarshani Premaratne
Ms. Nishalie Fernandopulle

Dr. Deluckshi Navaratnam

Lady Ridgeway Hospital for Children,

Colombo 8

Ethical Clearance

EMOTIONAL AND BEHAVIOURAL DIFFICULTIES OF CHILDREN AND ITS COMPARISON WITH PARENTAL STRESS DURING COVID-19 PANDEMIC

Principal Investigator: Dr. Deluckshi Navaratnam

Supervisor: Prof. Guwani Liyanage, Professor in Paediatrics, University of Sri Jayewardenepura

Study Design: Cross-sectional survey

Setting: Colombo District of Sri Lanka.

Period of study: 1st April to 30th September 2021

Funding: None

Dear Dr. Deluckshi Navaratnam,

We are pleased to inform you that ethical clearance has been granted for the above study.

Yours sincerely,

Dr. G. N. Lucas
Chairman, Ethical Review Committee, SLCP
